# Supplementary material for: The neural representation of absolute direction during mental navigation in conceptual spaces
Source: Commun Biol. 2021 Nov 16;4:1294. doi: 10.1038/s42003-021-02806-7 (PMC8595308; doi:10.1038/s42003-021-02806-7)
Supplement: Supplementary file 2 — Supplementary Material [file 42003_2021_2806_MOESM2_ESM.pdf]

The neural representation of absolute direction during mental navigation in conceptual spaces

Authors: Simone Viganò [1], Valerio Rubino [1], Marco Buiatti [1], Manuela Piazza [1]

Affiliations: [1]CIMeC, Center for Mind/Brain Sciences, University of Trento, Rovereto, Italy

Corresponding author: simone.vigano@unitn.it

SUPPLEMENTARY MATERIALS

Supplementary Figure 1
Supplementary Figure 2
Supplementary Figure 3

a

Nuisance regressors and exclusive mask

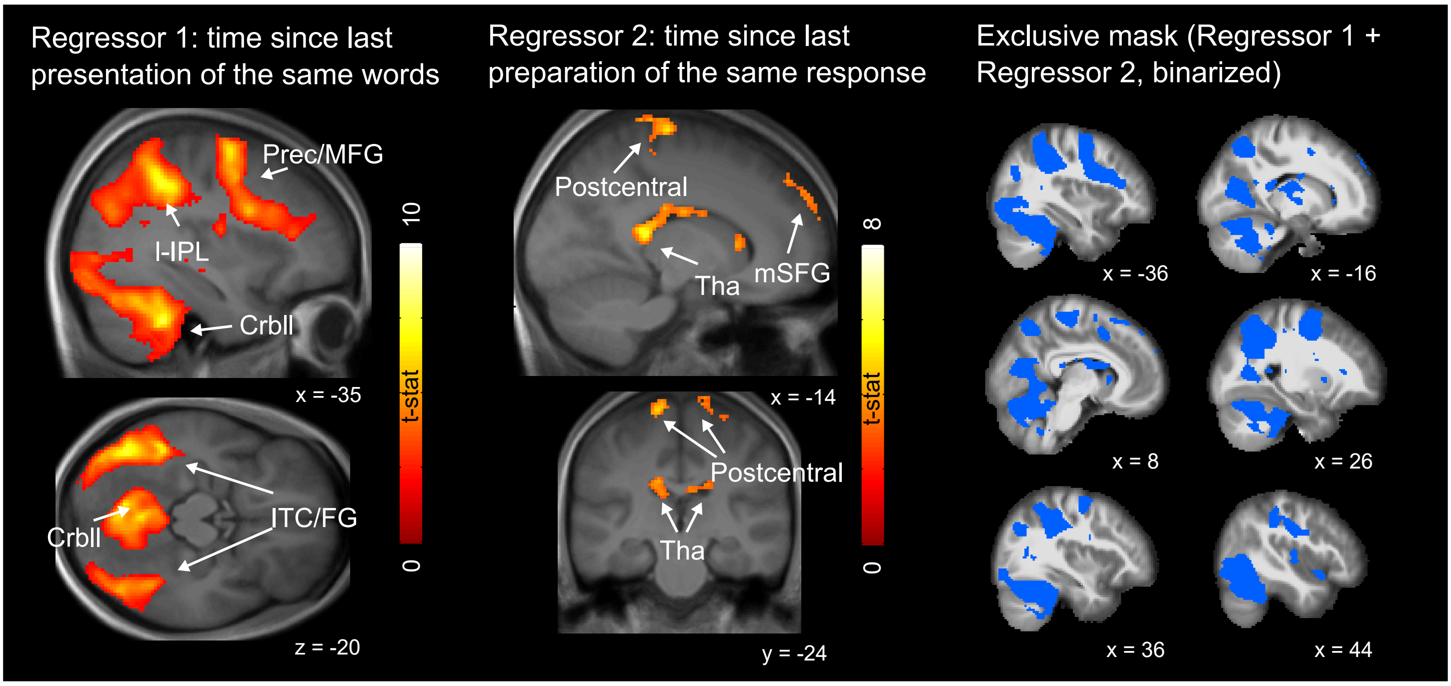

b

Adaptation to direction without controlling for confounding factors

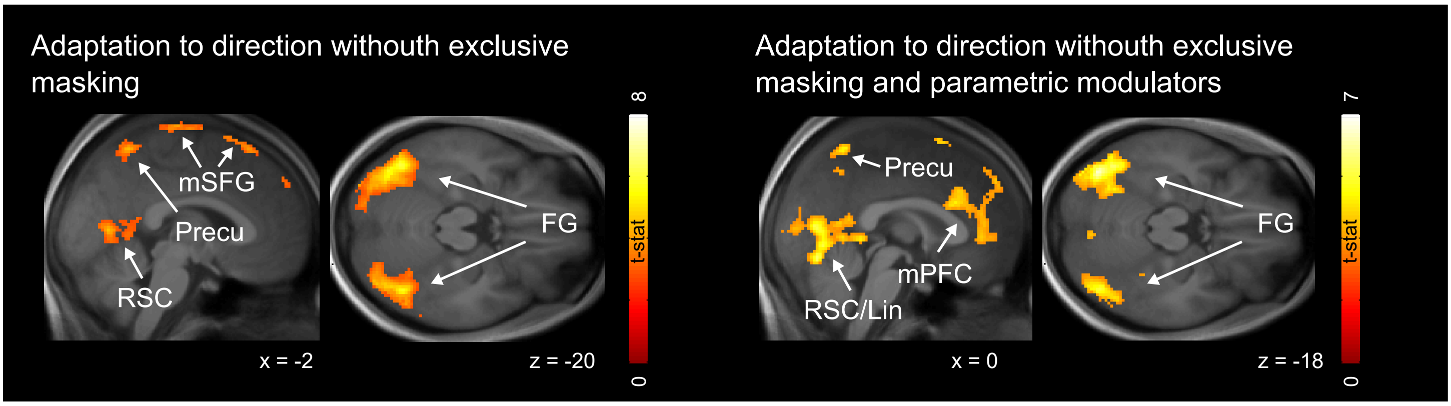

Supplementary Fig. 1 - Nuisance regressors and exclusive mask - a. We modelled the sequence of trials presented during each run taking into account, for each direction implied by a word pair, the elapsed time from the last presentation of the same pair (Regressor 1) and the last presentation of a word pair that required the same response preparation (Regressor 2). Results are thresholded at $p<.005$, FDR-corrected at cluster level with $q<.05$. The resulting activation maps were then binarized and combined to create an exclusive mask for our main analysis and to isolate brain regions that outside this network responded to directional adaptation. l-IPL = left Inferior Parietal Lobule; Prec = Precentral gyrus; MFG = Middle Frontal gyrus; Tha = Thalamus; Crbll = Cerebellum; ITC = Inferior Temporal Cortex; FG = Fusiform gyrus; mSFG = middle Superior Frontal gyrus**. b.** We report the results of our main analysis (fMRI adaptation to repetition of the same direction) without exclusive masking (left) and without both exclusive masking and the parametric modulators in the GLM for Regressors 1 and 2 (see Methods). Results are thresholded at $p<.005$, FDR-corrected at cluster level with $q<.05$. Precu = Precuneus; RSC = Retrosplenial cortex; Lin = Lingual gyrus; mSFG = Medial Superior Frontal gyrus; FG = Fusiform gyrus; Visual = Visual cortex.

a

No effect of cardinality on directional-adaptation

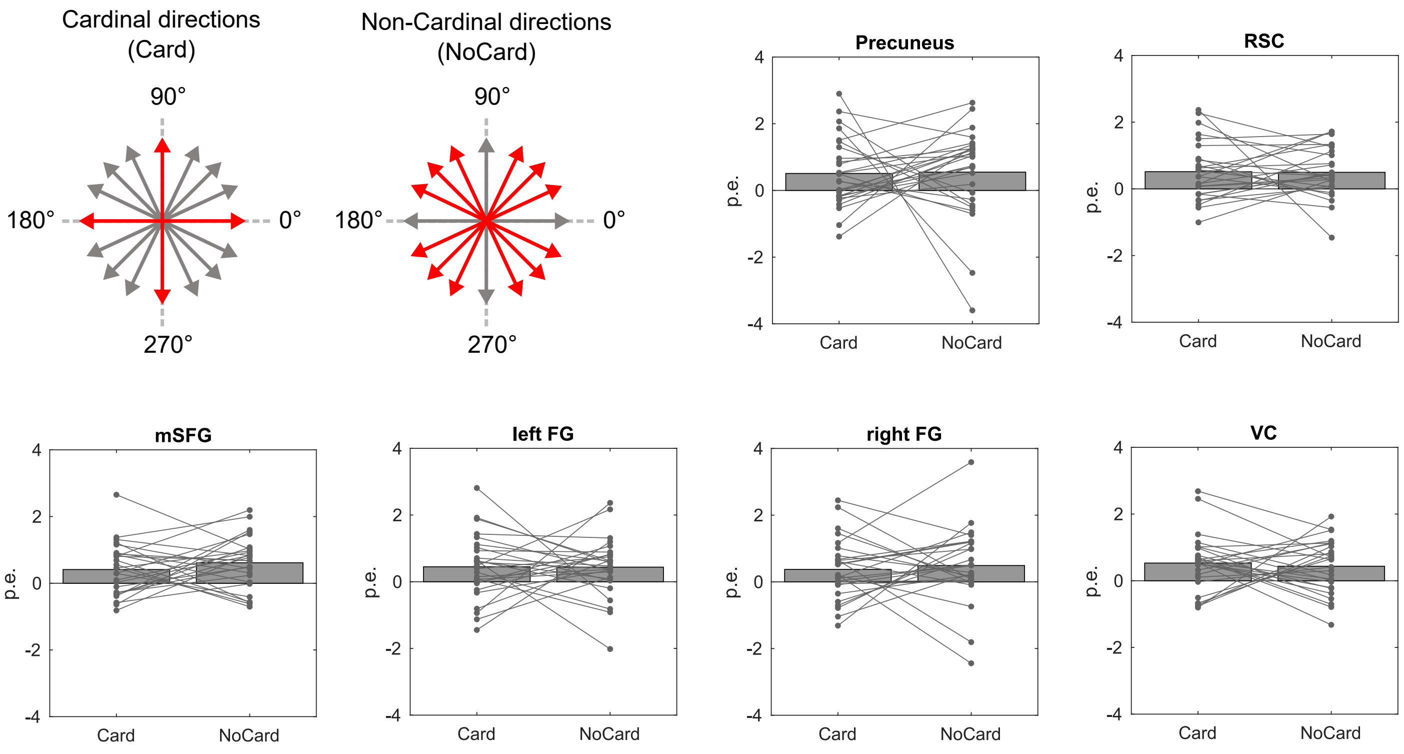

b

No effect of changes along size or pitch axes in direction-sensitive regions

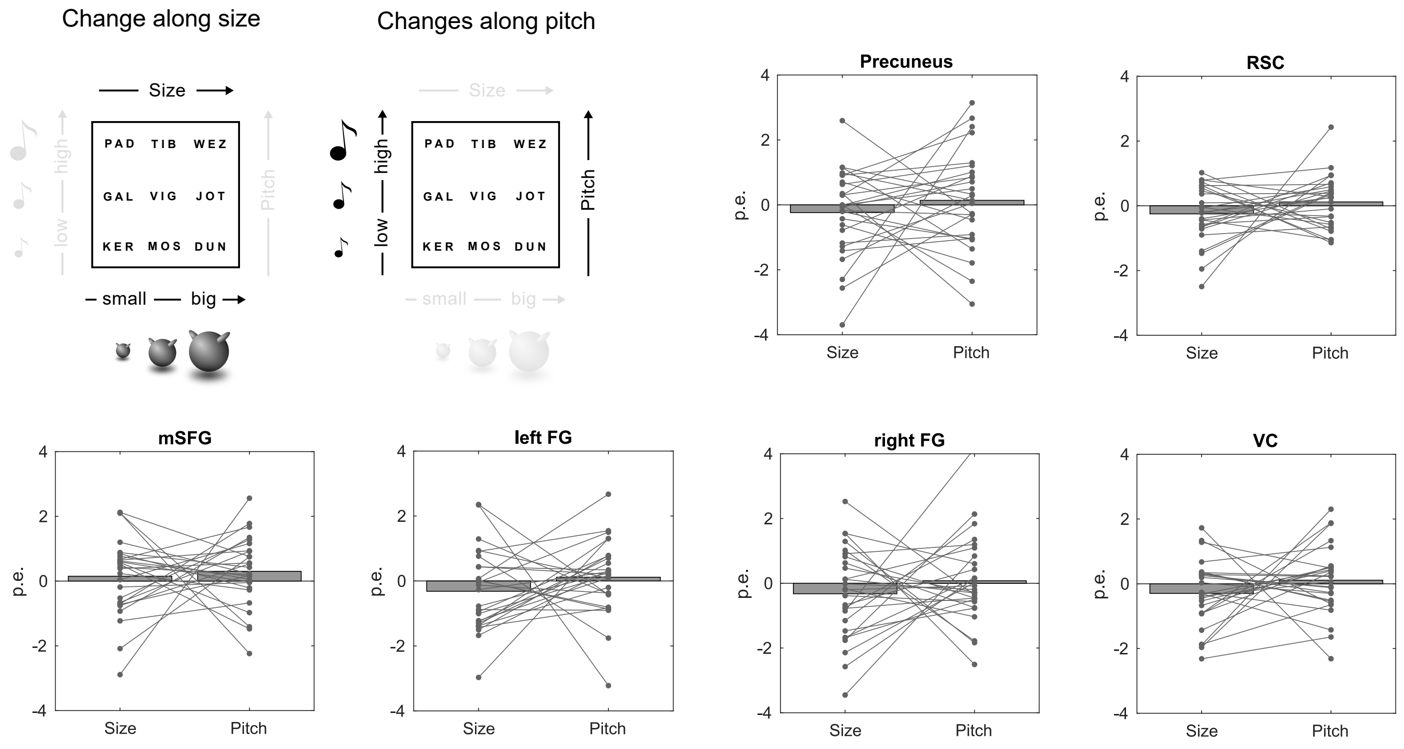

Supplementary Fig. 2 - No effect of cardinal direction or changes along size/pitch axes on directional-adaptation - a. We verified whether the directional-adaptation signal in the network of regions isolated by our main analysis was mostly driven by cardinal directions (0°, 90°, 180°, 270°). The cardinal (Card) vs non cardinal (NoCard) directions are represented in the top left panel. Parameter estimate of the direction-dependent adaptation effect for the two conditions is plotted for each region. Both cardinal and non cardinal directions elicited significant adaptation (all p-values <.05) in all these brain areas. Cardinal and non cardinal directions did not elicit a different adaptation effect (all p-values >.36). **b.** We additionally verified that brain regions showing adaptation to direction repetition were not modulated by changes along the individual axes of the feature space (size and pitch). None of the brain regions here isolated showed such an effect (all p-values > .14).

a

The correlation between word-pairs and direction repetition does not predict directional adaptation

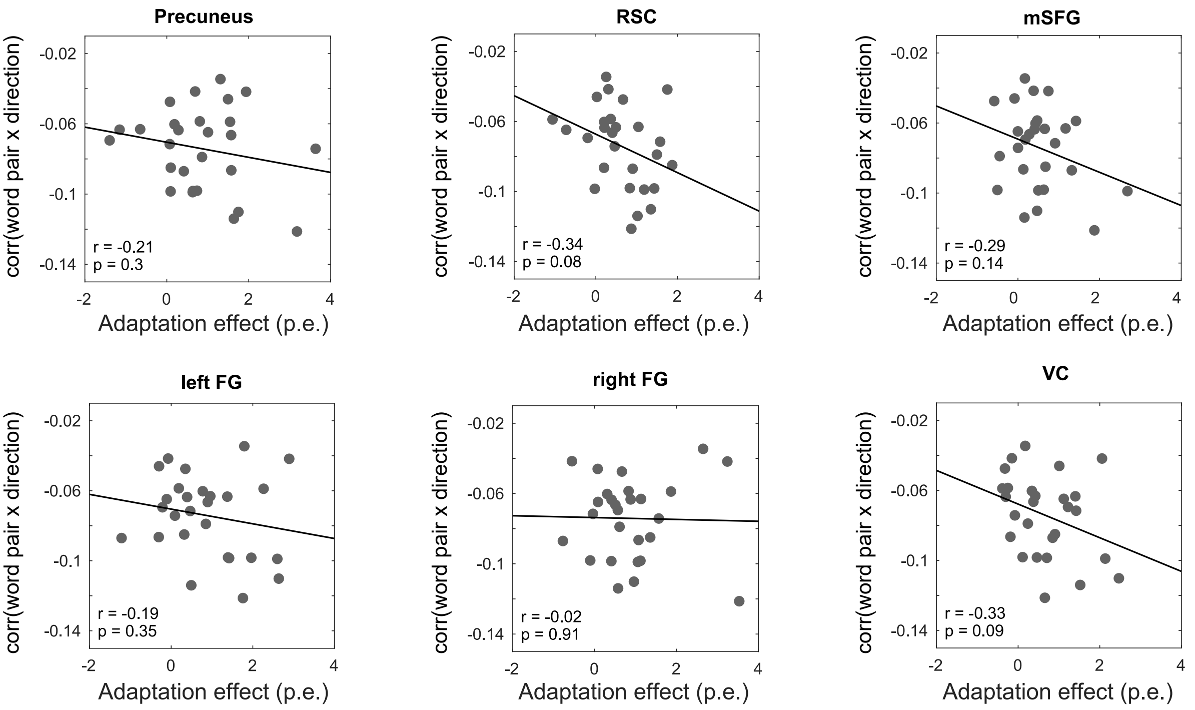

b

The correlation between response type and direction repetition does not predict directional adaptation

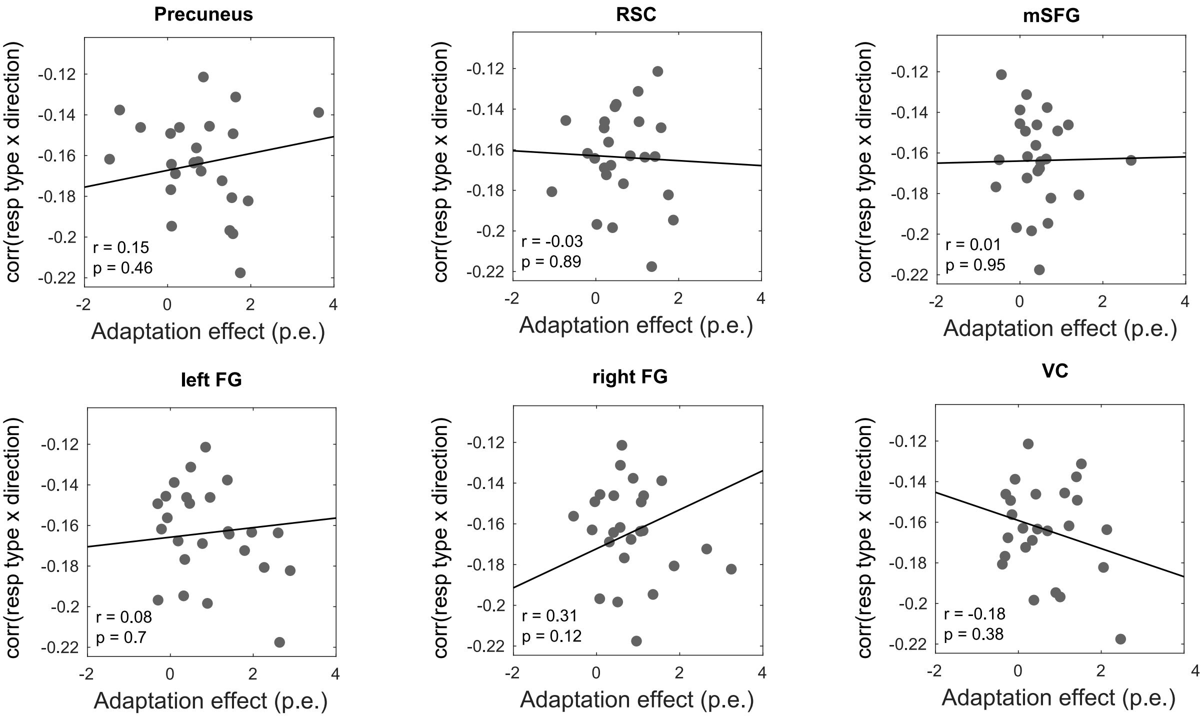

Supplementary Fig. 3 - Directional adaptation is not predicted by the degree of correlation between regressors - a. We verified whether the directional-adaptation signal in the network of regions isolated by our main analysis was correlated with the degree of collinearity between the regressor modelling repetition of word-pair and the one modelling repetition of direction. **b.** We verified whether the directional-adaptation signal in the network of regions isolated by our main analysis was correlated with the degree of collinearity between the regressor modelling the response type and the one modelling repetition of direction.
