# Supplementary material for: The neural representation of absolute direction during mental navigation in conceptual spaces
Source: Commun Biol. 2021 Nov 16;4:1294. doi: 10.1038/s42003-021-02806-7 (PMC8595308; doi:10.1038/s42003-021-02806-7)
Supplement: Supplementary file 3 — Description of Additional Supplementary Files [file 42003_2021_2806_MOESM3_ESM.pdf]

Description of Additional Supplementary Files

File name: Supplementary Data 1.
Description: Datasets for Fig. 2c, Supp Fig. 2a, Supp Fig. 2b, Supp Fig 3.
