# Supplementary material for: The neural representation of absolute direction during mental navigation in conceptual spaces
Source: Commun Biol. 2021 Nov 16;4:1294. doi: 10.1038/s42003-021-02806-7 (PMC8595308; doi:10.1038/s42003-021-02806-7)
Supplement: Supplementary file 5 — Reporting Summary [file 42003_2021_2806_MOESM5_ESM.pdf]

Corresponding author(s): Simone Viganò

Last updated by author(s): Oct 4 2021

Reporting Summary

Nature Research wishes to improve the reproducibility of the work that we publish. This form provides structure for consistency and transparency in reporting. For further information on Nature Research policies, see our Editorial Policies and the Editorial Policy Checklist.

Statistics

For all statistical analyses, confirm that the following items are present in the figure legend, table legend, main text, or Methods section.

n/a	Confirmed	
☐	☒	The exact sample size (n) for each experimental group/condition, given as a discrete number and unit of measurement
☐	☒	A statement on whether measurements were taken from distinct samples or whether the same sample was measured repeatedly
☐	☒	The statistical test(s) used AND whether they are one- or two-sided<br>*Only common tests should be described solely by name; describe more complex techniques in the Methods section.*
☐	☒	A description of all covariates tested
☐	☒	A description of any assumptions or corrections, such as tests of normality and adjustment for multiple comparisons
☐	☒	A full description of the statistical parameters including central tendency (e.g. means) or other basic estimates (e.g. regression coefficient) AND variation (e.g. standard deviation) or associated estimates of uncertainty (e.g. confidence intervals)
☐	☒	For null hypothesis testing, the test statistic (e.g. F, t, r) with confidence intervals, effect sizes, degrees of freedom and P value noted<br>*Give P values as exact values whenever suitable.*
☒	☐	For Bayesian analysis, information on the choice of priors and Markov chain Monte Carlo settings
☒	☐	For hierarchical and complex designs, identification of the appropriate level for tests and full reporting of outcomes
☒	☐	Estimates of effect sizes (e.g. Cohen's d, Pearson's r), indicating how they were calculated

Our web collection on statistics for biologists contains articles on many of the points above.

Software and code

Policy information about availability of computer code

Data collection	Matlab 2017b, SPM12
Data analysis	Matlab 2017b, SPM12

For manuscripts utilizing custom algorithms or software that are central to the research but not yet described in published literature, software must be made available to editors and reviewers. We strongly encourage code deposition in a community repository (e.g. GitHub). See the Nature Research guidelines for submitting code & software for further information.

Data

Policy information about availability of data

All manuscripts must include a data availability statement. This statement should provide the following information, where applicable:

- Accession codes, unique identifiers, or web links for publicly available datasets
- A list of figures that have associated raw data
- A description of any restrictions on data availability

In compliance with the rules of the Ethics Committee of the University of Trento, data are available from the corresponding author only for purposes related to the original research question (for which participants gave consent) and after the approval of the Ethics Committee itself.

Field-specific reporting

Life sciences study design

All studies must disclose on these points even when the disclosure is negative.

Sample size	Thirty-one right-handed students (21 female) from the University of Trento (Italy), participated in the experiment (mean age: 23.7, std: 3.2). The performance of all but four participants ceiled at over 90% of accuracy at the end of the training; the remaining four participants performed poorly (performance < 80% during the final comparison task) and were therefore excluded from the analyses. In total, 27 participants entered the final fMRI analysis, which tops previous similar studies investigating directional coding the field of spatial navigation (e.g., Baumann & Mattingley 2010; Kim & Maguire 2018; Marchette et al. 2015; Shine et al. 2016; Chadwick et al. 2015; Nau et al. 2020) and is in line with a priori estimation of sample size using G*Power (https://stats.idre.ucla.edu/other/gpower, assuming 80% power, alpha = 0.05 and medium effect size of d = 0.5, n = 27). The study was approved by the local Ethics Committee (Comitato Etico per la Sperimentazione con l'Essere Umano, University of Trento, Italy), and all participants gave written consent before the experiment.
Data exclusions	The performance of all but four participants ceiled at over 90% of accuracy at the end of the training; the remaining four participants performed poorly (performance < 80% during the final comparison task) and were therefore excluded from the analyses.
Replication	No replication was attempted
Randomization	We had only 1 experimental group so no randomization was necessary.
Blinding	No blinding procedure was implemented.

Reporting for specific materials, systems and methods

We require information from authors about some types of materials, experimental systems and methods used in many studies. Here, indicate whether each material, system or method listed is relevant to your study. If you are not sure if a list item applies to your research, read the appropriate section before selecting a response.

Materials & experimental systems

n/a	Involved in the study
☒	☐ Antibodies
☒	☐ Eukaryotic cell lines
☒	☐ Palaeontology and archaeology
☒	☐ Animals and other organisms
☐	☒ Human research participants
☒	☐ Clinical data
☒	☐ Dual use research of concern

Methods

n/a	Involved in the study
☒	☐ ChIP-seq
☒	☐ Flow cytometry
☐	☒ MRI-based neuroimaging

Human research participants

Policy information about studies involving human research participants

Population characteristics	Thirty-one right-handed students (21 female) from the University of Trento (Italy), participated in the experiment (mean age: 23.7, std: 3.2).
Recruitment	Participants were recruited through the university recruitment systems (web pages on social media)
Ethics oversight	The study was approved by the local Ethics Committee (Comitato Etico per la Sperimentazione con l'Essere Umano, University of Trento, Italy), and all participants gave written consent before the experiment.

Note that full information on the approval of the study protocol must also be provided in the manuscript.

Magnetic resonance imaging

Experimental design

Design type	Event-related
Design specifications	The experiment was organized in 8 runs of 48 trials each. There were 72 possible word pairs (pairs of the same word were excluded because they did not subtend any movement), each corresponding to one out of 16 different movement directions (assuming the x-axis as 0°, the possible direction of movements are 0°, 26.5°, 45°, 63.5°, 90°, 116.5°, 135°, 153.5°, 180°, 206.5°, 225°, 243.5°, 270°, 296.5°, 315°, 333.5° (Figure 1C)). Throughout the experiment, all 16 directions were sampled uniformly with 24 repetitions for each direction, equally divided between runs.
Behavioral performance measures	We recorded correct button press and we assessed performance by analysing mean and standard deviation.

Acquisition

Imaging type(s)	Functional and structural
Field strength	3T
Sequence & imaging parameters	TR = 1 sec; TE = 28 msec; FOV = 100mm; number of slices per volume = 65, acquired in interleaved ascending order; voxel size = 2 mm isotropic. T1-weighted anatomical images were acquired with an MP-RAGE sequence, with 1x1x1 mm resolution
Area of acquisition	whole brain

Diffusion MRI ☐ Used ☒ Not used

Preprocessing

Preprocessing software	SPM12
Normalization	classic normalization procedure as in the standard SPM12 pipeline
Normalization template	ICBM152
Noise and artifact removal	classic noise and artifact removal procedure as in the standard SPM12 pipeline, with regressors for motion corrections, realignment, tissue segmentation
Volume censoring	na

Statistical modeling & inference

Model type and settings	fmri adaptation
Effect(s) tested	We fit a GLM with SPM12 using a parametric modulator that, for each trial, predicted the fMRI signal as a function of the (log) time elapsed since the last presentation of the same direction (see Figure 2A for a simplified visualization). Group-level analyses were implemented as 2nd level analysis in SPM12

Specify type of analysis: ☐ Whole brain ☐ ROI-based ☒ Both

Anatomical location(s)	In order to verify whether or not the directional signal was present in the other regions of the brain where directional coding had been previously reported (e.g., entorhinal cortex, parahippocampus, subiculum, thalamus), we additionally applied this analysis following an ROI-based approach and selecting ROIs in the thalamus and parahippocampus (masks selected from Pickatlas (Maldjian et al. 2003)) as well as in the entorhinal cortex (mask selected from Maass et al. 2015) and in the subiculum (corresponding to Brodmann Area 27, selected from Pickatlas (Maldjian et al. 2003)).
Statistic type for inference (See Eklund et al. 2016)	Cluster-wise, FWE corr at q<.05
Correction	Cluster-wise, FWE corr at q<.05

Models & analysis

n/a	Involved in the study
☒	☐ Functional and/or effective connectivity
☒	☐ Graph analysis
☒	☐ Multivariate modeling or predictive analysis
